# Protocol for whole-mount immunofluorescence staining of ECM gel-embedded innervated pancreatic organoids

**DOI:** 10.1016/j.xpro.2024.103132

**Published:** 2024-06-13

**Authors:** Hüseyin Erdinç Beşikcioğlu, Ümmügülsüm Yurteri, Linhan Ye, Fang Zhang, Alessandra Moretti, Ibrahim Halil Gürcinar, Alper Dogruöz, Didem Karakas, Helmut Friess, Guralp Onur Ceyhan, Rouzanna Istvanffy, Ihsan Ekin Demir

**Affiliations:** 1 Department of Surgery, Klinikum rechts der Isar, Technical University of Munich, School of Medicine, Munich, Germany; 2 Department of Histology and Embryology, Faculty of Medicine, Hitit University, Çorum, Turkey; 3 Department of Internal Medicine I, Klinikum rechts der Isar, Technical University of Munich, School of Medicine, Munich, Germany; 4 Department of General Surgery, HPB-Unit, School of Medicine, Acibadem Mehmet Ali Aydinlar University, Istanbul, Turkey; 5 German Cancer Consortium (DKTK), Partner Site Munich, Munich, Germany; 6 CRC 1321 Modelling and Targeting Pancreatic Cancer, Klinikum rechts der Isar, Technical University of Munich, Munich, Germany; 7 Else Kröner Clinician Scientist Professorship for Translational Pancreatic Surgery, Technical University of Munich, Munich, Germany; 8 Department of Medical Biotechnology, Graduate School of Health Sciences, Acibadem University, Istanbul, Turkey

**Keywords:** Microscopy, Model Organisms, Organoids

## Abstract

The mandatory usage of extracellular matrix (ECM) gels in 3D cultures limits antibody penetration and increases background, while the removal of ECM gel causes disruption of morphology and sample loss. These factors pose challenges to effective immune labeling-based staining. Here, we present a protocol for whole-mount immunofluorescence staining of gel-embedded pancreatic organoids. We describe steps for sample fixation, blocking, and antibody incubation. We detail procedures for washing antibodies and mounting.

## Highlights

• Preparation of PBS-glycine buffer, IF-wash buffer, and fructose-glycerol clearing solution
• Steps for immunostaining of ECM gel-embedded innervated pancreatic organoids
• Steps for mounting samples with fructose-glycerol clearing solution

## Abstract (editor highlights)

Publisher’s note: Undertaking any experimental protocol requires adherence to local institutional guidelines for laboratory safety and ethics.

## Graphical abstract

**Figure:**
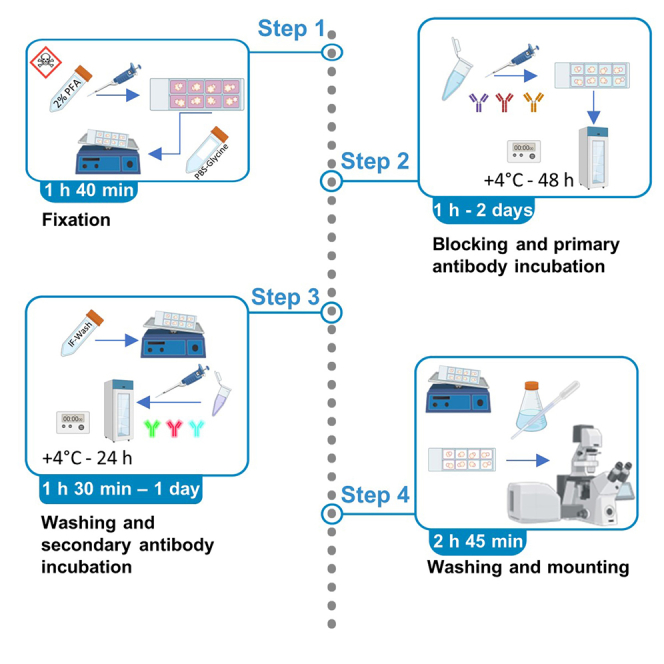

## Before you begin

We used this protocol for whole-mount immunofluorescence staining of innervated pancreas organoids. This protocol protects the morphology of inherent fragile structures such as axons and can be applied on other 3D culture settings.

***Note:*** The amount of described solutions are calculated for 8 well of 8 well chamber slide.

### Culture innervated pancreas organoids

**Timing: 7 days**

Culture innervated pancreas organoids in an 8-well chamber slide according to published protocol.^1^

### Preparations of stock solutions

**Timing: 30 min**

1. Prepare 100 mL of PBS-Glycine (10x) stock solution.
  a. Add 7.5 g of glycine in a 100 mL screw cap laboratory bottle.
  b. Add 90 mL of 10x PBS.
  c. Mix the solution on a magnetic stirrer until you get a homogenous solution.
  d. Adjust pH to 7.4.
  e. Complete the volume to 100 mL by adding 10x PBS.
  f. Check the pH whether it remains as 7.4 and adjust pH to 7.4 if it is needed.
  g. Filter through a 0.2 μm filter.
2. Prepare 100 mL of IF-Wash buffer (10x) stock solution.
  a. Add 0.5 g of NaN_3_ in a 100 mL screw cap laboratory bottle.
    **CRITICAL:** NaN_3_ is highly toxic. Avoid direct contact. Wear double gloves for thicker protection of skin and remove the gloves directly after handling NaN_3_. Do not forget wearing safety glasses, lab coat and closed-toe shoes.
  b. Add 1 g of BSA (Fraction V).
  c. Add 80 mL of 10x PBS.
  d. Add 2 mL of Triton X-100.
  e. Add 0.5 mL of Tween-20.
  f. Mix the solution on a magnetic stirrer until you get a homogenous solution.
  g. Adjust pH to 7.4.
  h. Complete the volume to 100 mL by adding 10x PBS.
  i. Check the pH whether it remains as 7.4 and adjust pH to 7.4 if it is needed.
  j. Filter through a 0.2 μm filter.

### Preparations of the buffers and (1X) solutions

**Timing: 15 min and 2 days**

3. Prepare fructose-glycerol clearing solution to use instead of fluorescence mounting media for improved transparency and preserving fluorescence signals.
  a. Add 33 mL of glycerol in a 100 mL screw cap laboratory bottle.
  b. Add 7 mL of dH_2_O.
  c. 29.72 g of fructose.
  d. Mix on a magnetic stirrer.

**CRITICAL:** It may take 2 days to get a homogeneous solution without any fructose crystals. Preparing this solution on the day starting the staining protocol is advised.

4. Prepare 4 mL 2% Paraformaldehyde solution.
  a. Mix 2 mL 4% Paraformaldehyde solution with 2 mL sterile PBS in a 15 mL conical tube.
  b. Warm up the 2% paraformaldehyde solution at 37°C.

**CRITICAL:** Paraformaldehyde is toxic. Do not forget to wear gloves and safety glasses. Use PFA under a fume hood and avoid inhaling and direct contact.

5. Prepare 50 mL PBS-Glycine solution.
  a. Add 5 mL PBS-Glycine (10X) stock solution in a 50 mL conical tube.
  b. Complete the volume to 50 mL with distilled water.
  c. Warm up the 1X PBS-Glycine solution at 37°C.
6. Prepare 50 mL (1X) IF-Wash buffer.
  a. Add 5 mL (10X) IF-Wash buffer stock solution in a 50 mL conical tube.
  b. Complete the volume to 50 mL with distilled water.
  c. Warm up the (1X) IF-Wash buffer at 37°C.
7. Prepare warm working-plate for keeping chamber slides warm during manipulation.
  a. Fill a T175 flask with sterile water.
  b. Add 3 drops of water bath sterilizer solution.
  c. Close the T175 flask with an unfiltered cap and seal with parafilm.
  d. Warm up the prepared working-plate at 37°C before starting any manipulation.

## Key resources table

**Table:** 

REAGENT or RESOURCE	SOURCE	IDENTIFIER
**Antibodies**		

Purified mouse anti-E-cadherin (dilution 1:250)	BD Biosciences	Cat#610182
Polyclonal rabbit glial fibrillary acidic protein (GFAP) (dilution 1:250)	Dako	Cat#Z0334
Chicken polyclonal β3-Tubulin (dilution 1:250)	Novus Biologicals	Cat#N100-1612
Goat anti-mouse Alexa Fluor 488 (dilution 1:500)	Thermo Fisher Scientific	Cat#A28175
Goat anti-rabbit Alexa Fluor 594 (dilution 1:500)	Thermo Fisher Scientific	Cat#A11037
Goat anti-chicken Alexa Fluor 647 (dilution 1:500)	Thermo Fisher Scientific	Cat#A21449

**Chemicals, peptides, and recombinant proteins**		

Phosphate-buffered saline	Sigma-Aldrich	Cat#P3813-10PAK
Paraformaldehyde Solution, 4% in PBS	Thermo Scientific	Cat#AAJ19943K2
Glycine	Sigma-Aldrich	Cat#410225
Sodium azide (NaN_3_)	Sigma-Aldrich	Cat#S2002
Bovine serum albumin fraction V	BSAV-RO Roche	Cat#10735094001
Triton X-100	Carl Roth	Cat#3051.3
Tween 20	Sigma-Aldrich	Cat#P1379-100ML
Glycerol	Carl Roth	Cat#3783.1
D(−)-fructose	Sigma-Aldrich	Cat#0127
Normal goat serum (10%)	Thermo Fisher Scientific	Cat#50197Z
DAPI (4′,6-diamidino-2-phenylindole, dihydrochloride)	Thermo Fisher Scientific	Cat#D1306

**Other**		

Syringe filter unit, 0.22 μm	Millipore	Cat#SLGSV255F
Horizontal shaker	N/A	N/A
Waterbath	N/A	N/A
Confocal microscope	Leica Microsystems	SP8

## Materials and equipment

**Table:** Fructose-glycerol clearing solution (1x)

Reagent	Final concentration	Amount
Glycerol	825 μL/mL	33 mL
dH_2_O	175 μL/mL	7 mL
Fructose	2.5 M	29.72 g
**Total Volume:**		**40 mL**

Fructose-glycerol clearing solution can be stored at +4°C up to 3 months.

**Table:** IF-wash buffer (10x) stock solution

Reagent	Final concentration (10x)	Final concentration (1x)	Amount
(10X) PBS	975 μL/mL	97.5 μL/mL	100 mL
NaN_3_	0.048 μg/mL	0.0048 μg/mL	0.5 g
BSA (Fraction V)	0.975 μg/mL	0.0975 μg/mL	1 g
Triton X-100	19.5 μL/mL	1.95 μL/mL	2 mL
Tween-20	4.878 μL/mL	0.4878 μL/mL	0.5 mL
**Total Volume:**			**102.5 mL**

IF-Wash buffer (10x) stock solution can be stored at +4°C up to 2 weeks.

**PBS/Glycine (10x) stock solution:** add 7.5 g glycine in 100 mL (10X) PBS.

PBS/Glycine (10x) stock solution can be stored at +4°C up to 2 weeks.

## Step-by-step method details

### Fixation and washing

**Timing: 1 h 40 min**

This step explains fixation of samples, washing the samples to clear any fixative residue and increasing permeabilization.

**CRITICAL:** Because the temperature directly affects the solidity of ECM gel, always keep your chamber slide on pre-warmed working-plate and warm up all buffers and solutions at 37°C. Please also consider performing all pipetting steps gently and slowly.

1. Fixation of samples.
  a. After 7 days of co-culturing, aspirate the medium from 8-well chamber.
  b. Add 500 μL of pre-warmed PBS in each well.
  c. Aspirate the PBS and treat each well with 500 μL of pre-warmed 2% PFA at R.T. for 15 min.

**CRITICAL:** The PFA is toxic. Do not forget to wear gloves and safety glasses. Use PFA under a fume hood and avoid inhaling and direct contact.

2. Washing samples for clearing fixative residue.
  a. Aspirate the PFA solution.
  b. Add 500 μL of pre-warmed 1x PBS-Glycine solution in each well.
  c. Wash samples for 10 min at RT on a horizontal shaker at 20 rpm.
  d. Aspirate the 1x PBS-Glycine solution and repeat steps 2.b. and 2.c. two more times (Totally 3 washing steps with 1x PBS-Glycine.).

**Pause point:** Samples can be stored in 1x PBS at +4°C up to 2 days before going on step 3.

3. Increasing permeabilization.
  a. Aspirate the PBS-Glycine solution.
  b. Add 500 μL of pre-warmed 1x IF-Wash buffer in each well.
  c. Rest the samples for 20 min at RT to increase permeabilization and antibody penetration.
  d. Aspirate the old 1x IF-Wash buffer.
  e. Add 500 μL of pre-warmed 1x IF-Wash buffer in each well.
  f. Continue the washing steps with pre-warmed 1x IF-Wash buffer for 10 min at RT on a horizontal shaker at 20 rpm.
  g. Repeat steps 3.d., 3.e. and 3.f two more times (Totally 4 washing steps with IF-Wash buffer. 1 time for 20 min and 3 times for 10 min).

### Blocking and primary antibody incubation

**Timing: 1 h and 2 days**

This step explains blocking for preventing non-specific binding and efficient incubation with primary antibody.

**CRITICAL:** Because the temperature directly affects the solidity of ECM gel, always keep your chamber slide on pre-warmed working-plate and warm up all buffers and solutions at 37°C. Please also consider performing all pipetting steps gently and slowly.

4. Blocking.
  a. Prepare 2.5 mL blocking solution as 2% Normal Goat Serum diluted in IF-Wash buffer.
    i. Mix 2 mL IF-Wash buffer and 500 μL 10% Normal Goat Serum in a 2.5 mL centrifuge tube.
    ii. Warm up the blocking solution at 37° in a water bath.
      ***Note:*** Normal goat serum is used for blocking according to the features of primary antibodies used in this study. Please consider the manufacturer’s recommendations of your primary antibodies. The serum of the species in which the secondary antibody is raised is also a good option for choosing the right blocking agent.
  b. Aspirate the IF-Wash buffer from the wells of chamber.
  c. Add 500 μL of 2% blocking solution which prepared at step 3.a. in each well and treat the samples for 1 h at room temperature.
5. Prepare the primary antibody mixture by diluting the primary antibodies as 1 to 250 ratio in IF-Wash buffer, while samples are being treated with blocking solution.
  ***Note:*** 300 μL of primary antibody mixture is needed for each well. It is recommended to prepare 2.5 mL antibody mixture for a whole 8-well chamber slide.
  a. Mix 10 μL of each primary antibody (listed below in Table 1) and 2470 μL IF-Wash buffer in a 2.5 mL centrifuge tube.
    **CRITICAL:** The indicated antibody combination is used in the same concentration (as 1 to 250) optimized for triple staining of Purified Mouse Anti-E-Cadherin, Polyclonal Rabbit Glial Fibrillary Acidic Protein (GFAP) and Chicken polyclonal β3-Tubulin. In the case of usage any other combinations do not forget to choose primary antibodies with different hosts and the optimization of antibody concentrations.
    ***Note:*** The secondary only control staining is advised for more reliable staining. One well of the sample should be filled with 300 μL IF-Wash buffer then be applied as same with the other wells during the rest of whole procedure.

**Table 1:** Primary antibodies

Primary antibody	Host	Concentration
Anti-E-Cadherin	Mouse	1 to 250
Anti-GFAP	Rabbit	1 to 250
Anti-β3-Tubulin	Chicken	1 to 250
6. After 1 h of incubation, remove the blocking solution and add 300 μL of primary antibody mixture in each well.
  a. Close the cap of chamber slide and seal with parafilm to prevent evaporation.
  b. Incubate at +4°C degree for 2 days.

### Washing and secondary antibody incubation

**Timing: 1 h 30 min and 1 day**

This step explains clearing primary antibody residue and efficient incubation with secondary antibody.

**CRITICAL:** Because the temperature directly affects the solidity of ECM gel, always keep your chamber slide on pre-warmed working-plate during the procedure and warm up all buffers and solutions at 37°C before start using. Please also consider performing all pipetting steps gently and slowly.

7. Take the samples out from the fridge and let them sit at RT for at least 1 h to make ECM gel more stable.

**CRITICAL:** It is very important to warm samples at room temperature for obtaining more stable ECM gel before any further application. Otherwise, samples can be easily lost during washing steps.

8. Aspirate the primary antibody mixture from the wells.
  a. Add 500 μL of pre-warmed 1x IF-Wash buffer in each well.
  b. Treat the samples with pre-warmed 1x IF-Wash buffer for 10 min at RT on a horizontal shaker at 20 rpm.
  c. Aspirate the old 1x IF-Wash buffer.
  d. Add 500 μL of pre-warmed 1x IF-Wash buffer in each well.
  e. Continue the washing steps with pre-warmed 1x IF-Wash buffer for 10 min at RT on a horizontal shaker at 20 rpm.
  f. Repeat steps 8.c., 8.d. and 8.e. one more time.
9. Prepare the secondary antibody mixture by diluting the secondary antibodies (listed below in Table 2) as 1 to 500 ratio in IF-Wash buffer.
  ***Note:*** 300 μL of secondary antibody mixture is needed for each well. It is recommended to prepare 2.5 mL antibody mixture for a whole 8-well chamber slide.
  a. Mix 5 μL of each secondary antibodies, 2.5 μL DAPI for nuclear staining and 2482.5 μL IF-Wash buffer in a 2.5 mL centrifuge tube.
    **CRITICAL:** The given secondary antibody combination and concentrations are optimized according to the features of selected primary antibodies in this experiment. Other options should be tried out if the primary antibody combination is changed.

**Table 2:** Secondary antibody mixture

Secondary antibody	Reactivity	Host	Concentration
Alexa Fluor 488	Mouse	Goat	1 to 500
Alexa Fluor 594	Rabbit	Goat	1 to 500
Alexa Fluor 647	Chicken	Goat	1 to 500
Nuclear staining	Stock concentration		
DAPI	10x		1 to 1000
10. Aspirate the IF-Wash buffer from the wells and add 300 μL of the secondary antibody mixture in each well.
11. Incubate the samples at +4°C degree for 24 h.

### Washing and mounting

**Timing: 2 h 45 min**

This last step includes excessive washing steps for completely clearing secondary antibody residue and mounting samples with fructose-glycerol clearing solution which is also make samples more transparent.

**CRITICAL:** Because the temperature directly effects the solidity of ECM gel, always keep your chamber slide on pre-warmed working-plate during the procedure and warm up all buffers and solutions at 37°C before start using. Please also consider performing all pipetting steps gently and slowly.

12. Take the samples out from the fridge and let them sit at RT for at least 1 h to make ECM gel more stable.

**CRITICAL:** It is very important to warm samples at room temperature for obtaining more stable ECM gel before any further application. Otherwise, samples can be easily lost during washing steps.

13. Wash the samples with 1x IF-Wash buffer.
  a. Aspirate the secondary antibody mixture from the wells.
  b. Add 500 μL of pre-warmed 1x IF-Wash buffer in each well.
  c. Treat the samples with pre-warmed 1x IF-Wash buffer for 30 min at RT on a horizontal shaker at 20 rpm.
  d. Aspirate the old 1x IF-Wash buffer.
  e. Add 500 μL of pre-warmed 1x IF-Wash buffer in each well.
  f. Continue the washing steps with pre-warmed 1x IF-Wash buffer for 20 min at RT on a horizontal shaker at 20 rpm.
  g. Repeat steps 9.c., 9.d. and 9.e. two more time (Totally 4 washing with 1x IF-Wash buffer. 1 time for 30 min and 3 times for 20 min).
14. Wash the samples with 1x PBS buffer.
  a. Aspirate IF-Wash buffer from the wells.
  b. Add 500 μL of pre-warmed 1x PBS in each well.
  c. Treat the samples with pre-warmed 1x PBS buffer for 20 min at RT on a horizontal shaker at 20 rpm.
  d. Aspirate the old 1x PBS.
  e. Add 500 μL of pre-warmed 1x PBS in each well.
  f. Continue the washing steps with pre-warmed 1x PBS buffer for 20 min at RT on a horizontal shaker at 20 rpm.
  g. Repeat steps 10.d., 10.e. and 10.f. two more time (Totally 4 washing with 1x PBS).
15. Mounting of the samples with fructose-glycerol solution.
  a. Aspirate the PBS from the wells.
  b. Add 50–100 μL of fructose-glycerol clearing solution until samples are completely covered with the fructose-glycerol clearing solution.
    **CRITICAL:** The fructose-glycerol cleaning solution is semi viscous that may cause air bubbles. Warming up the fructose-glycerol cleaning solution up to 37°C and using plastic Pasteur pipettes for transfer help to reduce bubbling.
  c. The samples will be ready for imaging after storage at +4°C for 24 h.
    ***Note:*** Samples can be stored at +4°C for 4 weeks and at −20°C for several months. The frozen samples can be thawed at 37°C to minimize possible effects of crystallization.
    ***Note:*** In this study, Samples were imaged with confocal microscopy (Leica SP8) at following settings (Data S1): Magnification: 20x. Resolution: 1024 × 1024 px. Scan speed: 700 hz. Z-Step size: 2 μm. Laser Line (405 nm): Intensity: 1.0023%. Laser Line (499 nm) Intensity: 3.0012%, Laser Line (598 nm) Intensity: 0.3999%, Laser Line (653 nm) Intensity: 1.0005%. Sensor for 405 nm: PMT 4. Sensor for 499 nm, 598 nm and 653 nm: HyD Gate Start: 0.3 ns, Gate End: 6 ns. Images were processed with Leica Application Suite X software.

## Expected outcomes

3D culture models such as organoids provide the advantages of including different cell types and enabling cell – cell and cell – ECM interactions. Visualization of ECM-embedded 3D cultures is very important for accurate histopathological evaluations. However, especially the immune labeling-based staining is a real challenge for the scientists working in this field. The limitations mainly arise from the presence of ECM gel. The ECM gel (with regarding to ECM stiffness) reduces the antibody penetration and induces the background autofluorescence. These issues can be minimized by clearing away the ECM gel.^2^ Cryosectioning is also an alternative for immunostaining of 3D ex-vivo models and their inner structures.^3^^,^^4^ However, morphological disruption is inevitable in both techniques. Due to the removal of ECM gel, organoids tend to aggregate, bigger organoids collapse, and sample is lost during washing steps. It is almost impossible to protect the inherent fragile structures such as axons. Cryosectioning limits the volumetric imaging and the neurite tracing in a wide area by causing deformation on the tissue wholeness. This step-by-step protocol describes a whole-mount immunofluorescence staining method for innervated pancreas organoids without the ECM gel removal. The fixation is modified by reducing fixation time to 15 min with diluted PFA as 2%. The PBS modified with Glycine and IF-Wash buffer decrease the non-specific binding. Optimized fixation, modified buffers and increased washing steps decrease the fluorescence background. Keeping the ECM gel takes the advantages of the protection of neural and organoid morphology. A more accurate software-based investigation of the neural plasticity in pancreatic cancer is also possible with the confocal microscopic images of the samples. This protocol is optimized for Matrigel embedded innervated pancreas organoids and anticipated to be applicable to other ECM-embedded 3D culture models.

**Figure 1:**
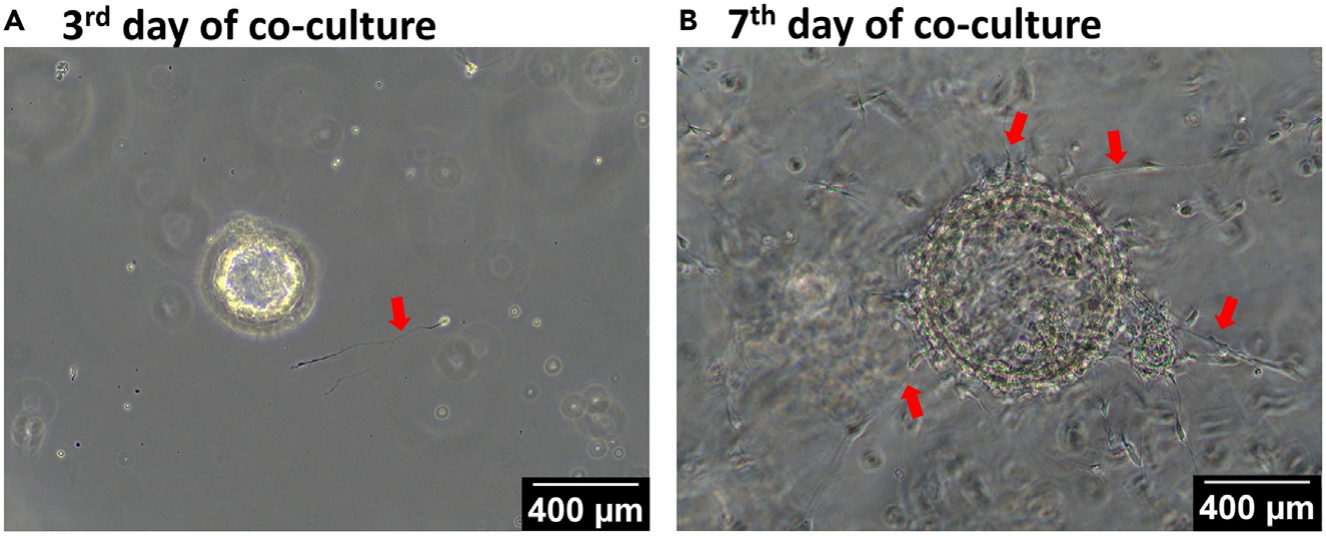
The development of innervated pancreas organoids in co-cultures Neuronal morphology on the 3rd day of co-culture (A) Phase-contrast, 10X, Scale Bar: 400 μm). Grown neurons with better initiation with pancreas organoids on the 7th day of co-culture (B) Phase-contrast, 10X, Scale Bar: 400 μm). Arrows shows the neurons in both picture (→).

After 7 days of co-culturing with neural cells and organoids, neuronal morphology can be observed on the 3rd day of co-culturing (Figure 1A) with bright-field microscopy. After 7 days of co-culturing, innervated pancreas organoids are ready for fixation and whole-mount staining (Figure 1B).

**Figure 2:**
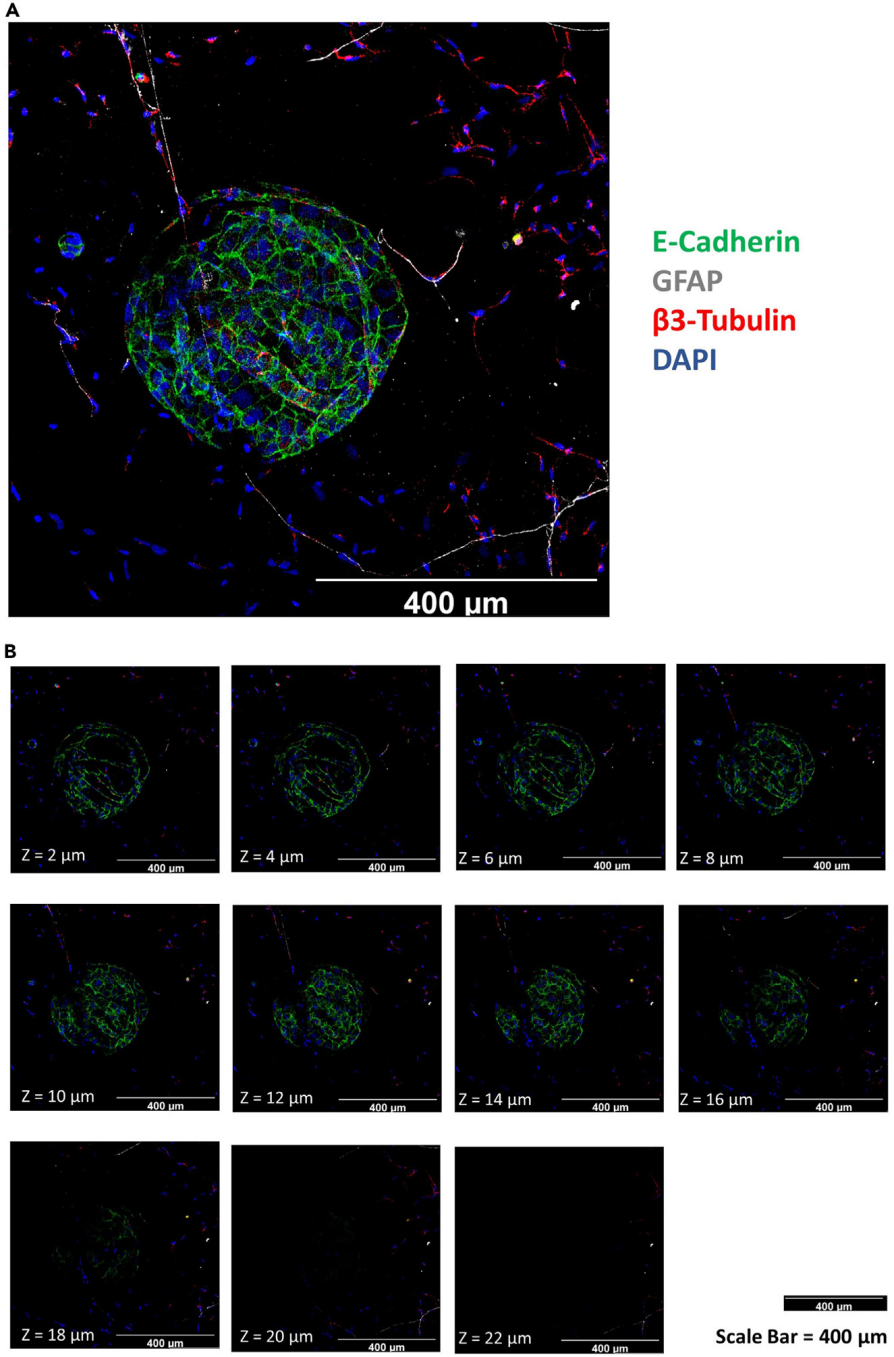
Whole-mount immune fluorescence staining of innervated pancreas organoids and Z-steps E-Cadherin positive (Green) pancreas organoid at the center of the image. β3-Tubulin (Red) and GFAP (Gray) positive neurons located around the organoid. The integrity of the neural projections is preserved, and they extend toward the organoid. Nuclei were stained with DAPI (Blue). Maximum projection of Z-stack (A) and single projection images of each Z-step (B) (Confocal microscopy, 20X, Z-Step Size = 2 μm, Scale Bar: 400 μm).

**Figure 3:**
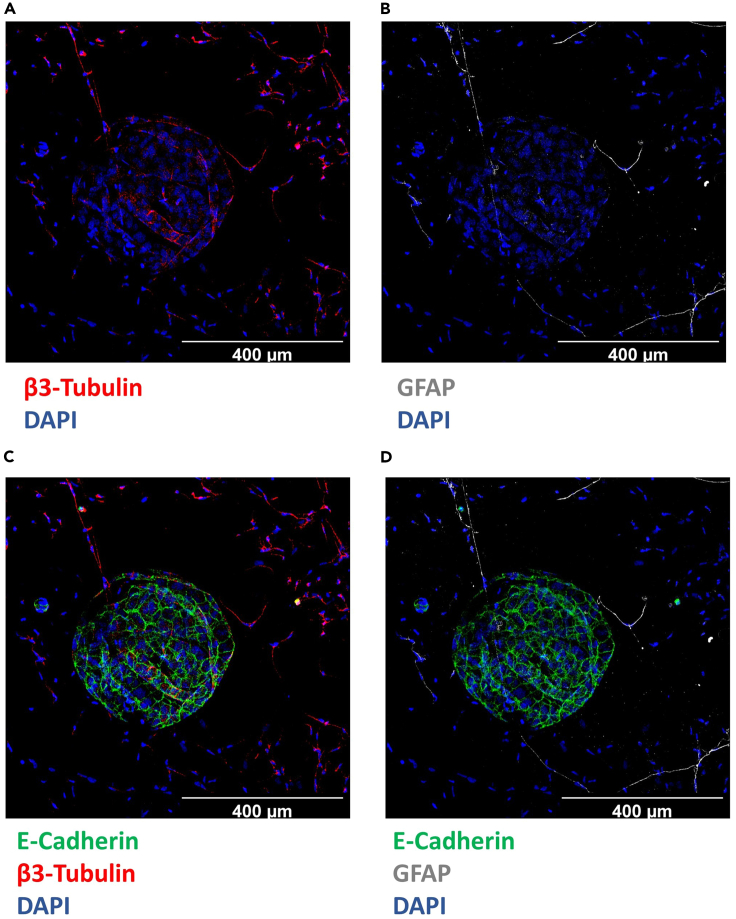
Whole-mount immune fluorescence staining of innervated pancreas organoids β3-Tubulin positive neurons (Red) and nuclei stained with DAPI (Blue) (A) GFAP positive myelin sheets of axons surrounded by Schwann cells (Gray) and nuclei stained with DAPI (Blue) (B). E-Cadherin positive pancreas organoid (Green), β3-Tubulin positive neurons (Red) and nuclei stained with DAPI (Blue) (C) E-Cadherin positive pancreas organoid (Green), GFAP positive myelin sheets of axons surrounded by Schwann cells (Gray) and nuclei stained with DAPI (Blue) (D) (Confocal microscopy, 20x, Scale Bar: 400 μm).

This protocol makes it possible to clearly image the innervation in pancreas organoids via confocal microscopy. The integrity of neuronal projections is preserved and their interactions with the pancreas organoids can be seen (Figures 2 and 3). Furthermore, 3D animations and 3D software-based analysis can be performed with Z-stack images in several sections taken by confocal microscopy (Methods video S1).

Methods video S1. 360° rotating video of innervated pancreas cancer organoid E-Cadherin positive pancreas organoid (Green), β3-Tubulin positive neurons (Cyan) and nuclei stained with DAPI (Blue). (Confocal microscopy, 20x), related to expected outcomes.

**Figure 4:**
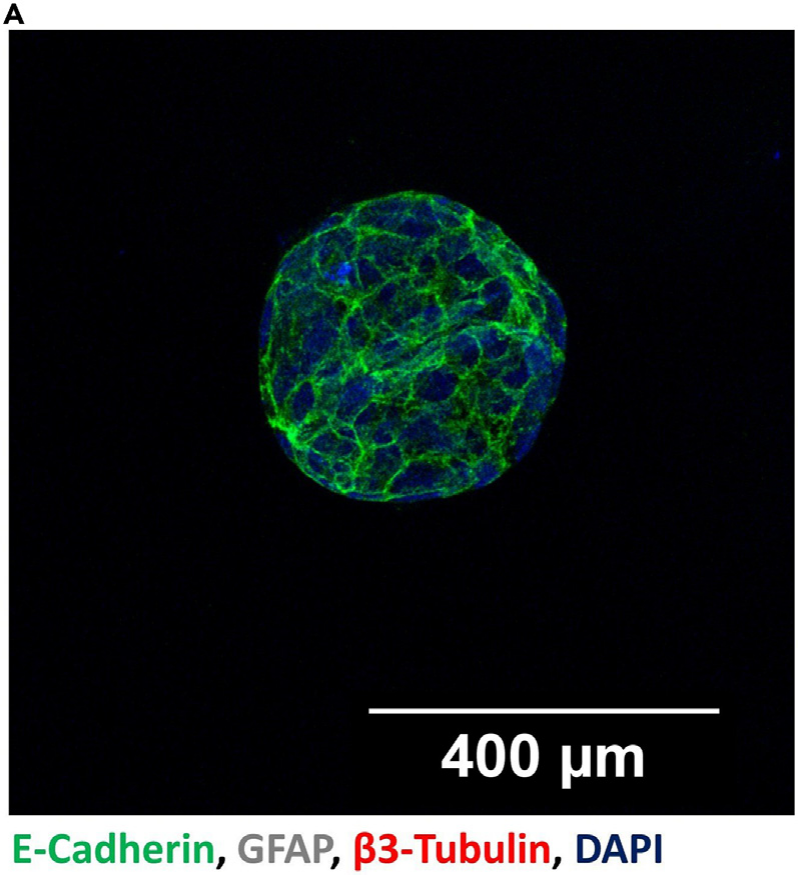
Whole-mount immune fluorescence staining of non-innervated pancreas organoids Non-innervated normal mouse pancreas organoid is positive for E-Cadherin (Green) but negative for β3-Tubulin (Red) and GFAP (Gray) (A) (Confocal microscopy, 20x, Scale Bar: 400 μm).

**Figure 5:**
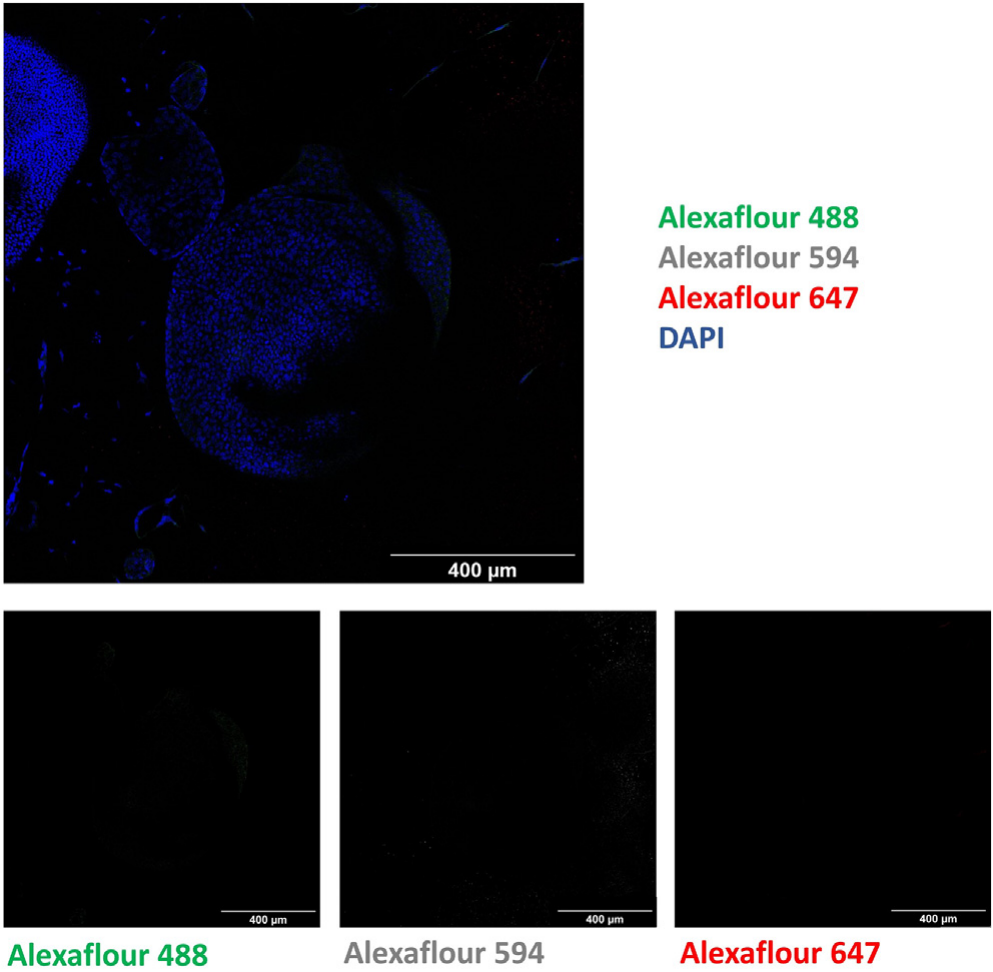
Control whole-mount immune fluorescence staining of innervated pancreas organoids by using only secondary antibodies and DAPI A smooth autofluorescence is observed upper right edge of the organoid for Alexa Flour 488 (Green), A little fluorescence particles are observed for Alexa Flour 594 (Gray) and Alexa Flour 647 (Red) (A) (Confocal microscopy, 20x, Scale Bar: 400 μm).

Non-innervated normal pancreas organoids (derived from monoculture of pancreas organoids) can be used as a negative control. There should not be any signal for neuronal marker β3-Tubulin and glial marker GFAP (Figure 4). The control staining with only secondary antibodies should not present any fluorescence signal (Figure 5).

## Limitations

Although, this protocol provides an approach to maintain morphology of extremely fragile structures, low antibody penetration and autofluorescence background can be still observed depending on ECM gel stiffness and cell population. The antibody penetration to inner sides of the organoids also reduces in correlation with the increasing organoid size. The bigger organoids (perimeter >3 mm) are more difficult for immune-labeling and imaging. This method is effective on labeling cytoplasmic and membrane structures, but antibody penetration cannot be sufficient for nuclear structures.

## Troubleshooting

### Problem 1

Sample loss during the fixation, washing steps and manipulations of the samples.

### Potential solution

• The ECM gel is dissolved when the temperature is around +4°C and can be discarded during manipulations of the samples. Please consider that all the samples are warmed up to room temperature and all the solutions at 37°C (Please consider during whole procedure).
• PFA decreases the stiffness of the ECM gel. Tilt the chamber slide at 45° angle and samples gently add PFA and any other buffers on the bottom corners of the wells. This technique also helps prevent the detachment of the gel (Please consider during all washing steps).

### Problem 2

The antibody penetration is not sufficient.

### Potential solution

• The longer incubation time (up to 3 days at +4°C) and with the primary antibody mixture enables the binding to surface proteins (such as E-cadherin) and cytoskeletal proteins (such as β3-Tubulin) (Please see Step 6/b).
• The antibody penetration can be negatively affected if the cell population is too much (e.g., more than 40∗10^3^ cell/mL in 25 μL ECM gel) in one ECM gel droplet. Lower sample density allows better antibody penetration (Please see the culture innervated pancreas organoids and referred protocol^1^ in before you begin section).
• The size of the organoids also causes lower antibody penetration. If the experimental plan does not need a longer period, try to fix samples with 2% PFA before their perimeter become bigger than 3 mm (Please see Step 1).
• The less volume of ECM gel helps for better antibody penetration. Reduce the volume of ECM gel droplets as 20 μL in your culture setup. Do not forget to redefine cell numbers according to ECM gel volume (Please see the culture innervated pancreas organoids and referred protocol^1^ in before you begin section).

### Problem 3

Autofluorescence background.

### Potential solution

• The aldehyde containing fixatives such as PFA increase the autofluorescence.^5^ On the other hand, using methanol as a fixative causes organoid to shrink and is not suitable for 3D cultures. Determination of the minimum fixation time which is enough for the fixation of samples helps to avoid autofluorescence. Limiting the fixation time to 15 min is sufficient for fixation with less autofluorescence for this protocol. The smaller samples can be fixed in less time. The fixation time can be reduced according to sample size (Please see Step 1).
• Glycine tends to bind free aldehyde groups. Therefore, increasing the first washing steps with 0.1 M PBS-Glycine solution can reduce PFA-caused autofluorescence background^6^ (Please see Step 2).

## Resource availability

### Lead contact

Further information and requests for resources and reagents should be directed to and will be fulfilled by the lead contact, Ihsan Ekin Demir (ekin.demir@tum.de).

### Technical contact

Further information and requests for any technical issue should be directed to and will be fulfilled by the technical contact, Huseyin Erdinc Besikcioglu (erdincbesikcioglu@gmail.com).

### Materials availability

This study did not generate new unique reagents.

### Data and code availability

This study did not generate datasets or codes.

## Supplemental information

Data S1. Detailed settings of confocal microscope, related to step 15
