# Supplementary material for: Protocol for whole-mount immunofluorescence staining of ECM gel-embedded innervated pancreatic organoids
Source: STAR Protoc. 2024 Jun 13;5(2):103132. doi: 10.1016/j.xpro.2024.103132 (PMC11227007; doi:10.1016/j.xpro.2024.103132)
Supplement: Data S1. Detailed settings of confocal microscope, related to step 15 [file mmc1.pdf]

Image: **Region 3_Merged_Crop001**

Size: **166,11 MB**

File Location: **C:\Users\erdin\OneDrive\My Articles\Jove_2022\RAW Jove figures\lasx\11.11.2020_kpc1510+2d b3 gfap.lif**

Start Time: **11.11.2020 15:32:02.107**

End Time: **11.11.2020 15:31:43.831**

Total Exposures: **44 (4 channels, 11 frames)**

Data from: **LAS X 3.5.5.19976**

Shown Devices: Active and Inactive (Toggle)

Dimensions

Dimension	Logical Size	Physical Length	Start Position	End Position	Pixel Size / Voxel Size
X	1953	869.87 µm	0 µm	869.87 µm	0.446 µm
Y	1933	860.96 µm	0 µm	860.96 µm	0.446 µm
Z	11	20 µm	68 µm	87.99 µm	2 µm

Channels

LUT	Resolution	Min	Max	STED: DetectorMode / Huygens saturation factor / Wavelength
Blue	8	0	255	--- / --- / ---
Green	8	0	255	--- / --- / ---
Red	8	0	255	--- / --- / ---
Cyan	8	0	255	--- / --- / ---

Time Stamps:

Frame (Show All)	Relative Time (s)	Absolute Time (h:m:s.ms)	Date
1	0,000	15:32:02.107	11.11.2020
44	-18,276	15:31:43.831	11.11.2020

Confocal Settings

Name	Value
Rotator Angle	-1.38 °
Scan Mode	xyz
Scan Direction X	Bidirectional
Scan Speed	700 Hz
Version Number	15
StagePosX	50,213.1 µm
StagePosY	47,345.75 µm
ZPosition	8.77 µm
IsSuperZ	0
Magnification	20

ObjectiveName	HC PL APO CS2 20x/0.75 IMM
Immersion	IMM
Numerical Aperture	0.75
RefractionIndex	1.518
Zoom	1.28
Pinhole	56.6 µm
PinholeAiry	999.82 mAU
EmissionWavelength for PinholeAiry Calculation	580 nm
FrameAverage	1
LineAverage	1
FrameAccumulation	1
Line_Accumulation	1
IsUserSettingNameSet	0
IsRoiScanEnable	0

Filter Wheels / Other Motorized Devices

**Device Name**	**Filter Name/Position**
Target Slider	Target Park
X2 Lens Changer	CS2 UV Optics 2

Lasers

**LaserName**	**OutputPower**
**Diode 405**	**On**
**WLL**	**On, 70,0000 %**

Laser Lines

**Laser Line**	**Intensity**
(405 nm)	Shutter: on, Intensity: 1.0023%
(499 nm)	Shutter: off, Intensity: 0.0000%
(598 nm)	Shutter: off, Intensity: 0.0000%
(653 nm)	Shutter: off, Intensity: 0.0000%
(470 nm)	Shutter: off, Intensity: 0.0000%
(471 nm)	Shutter: off, Intensity: 0.0000%
(670 nm)	Shutter: off, Intensity: 0.0000%
(669 nm)	Shutter: off, Intensity: 0.0000%
(668 nm)	Shutter: off, Intensity: 0.0000%

Detectors

**Name**	**Channel**	**Type**	**Location**	**Active**	**Gain**	**Offset**	**Gate Start**	**Gate End**	**Gate Ref. Wavelength**
HyD 1	Channel 1	HyD (395nm - 400nm) Standard mode	Internal	Inactive	100	--	-- Time Gating not activated --		
PMT 2	Channel 2	PMT (400nm - 405nm)	Internal	Inactive	0	0	-- Time Gating not supported --		
HyD 3	Channel 3	HyD (405nm - 410nm) Standard mode	Internal	Inactive	100	--	-- Time Gating not activated --		
PMT 4	Channel 4	PMT (417nm - 462nm)	Internal	Active	800	-0.5	-- Time Gating not supported --		
	Transmission						-- Time Gating not supported --		

PMT Trans	Channel	PMT	TLD	Inactive	235.2	0	

Sequential Setting Nr.1

Pinhole	56.6 µm
PinholeAiry	999.82 mAU
EmissionWavelength for PinholeAiry Calculation	580 nm
Target Slider	Target Park
X2 Lens Changer	CS2 UV Optics 2
HyD 1 (395nm - 400nm) Standard mode	Inactive, Gain: 100, Offset: 0
HyD Timegate (-1 nm)	Inactive, Gate Start: 0.3 ns, Gate End: 6 ns
PMT 2 (400nm - 405nm)	Inactive, Gain: 0, Offset: 0
HyD 3 (405nm - 410nm) Standard mode	Inactive, Gain: 100, Offset: 0
HyD Timegate (-1 nm)	Inactive, Gate Start: 0.3 ns, Gate End: 6 ns
PMT 4 (417nm - 462nm)	Active, Gain: 800, Offset: 0
PMT Trans	Inactive, Gain: 235, Offset: 0
Laser Line (405 nm)	Shutter: on, Intensity: 1.0023%
Laser Line (499 nm)	Shutter: off, Intensity: 0.0000%
Laser Line (598 nm)	Shutter: off, Intensity: 0.0000%
Laser Line (653 nm)	Shutter: off, Intensity: 0.0000%
Laser Line (470 nm)	Shutter: off, Intensity: 0.0000%
Laser Line (471 nm)	Shutter: off, Intensity: 0.0000%
Laser Line (670 nm)	Shutter: off, Intensity: 0.0000%
Laser Line (669 nm)	Shutter: off, Intensity: 0.0000%
Laser Line (668 nm)	Shutter: off, Intensity: 0.0000%

Sequential Setting Nr.2

Pinhole	56.6 µm
PinholeAiry	999.82 mAU
EmissionWavelength for PinholeAiry Calculation	580 nm
Target Slider	Target Park
X2 Lens Changer	CS2 UV Optics 2
HyD 1 (509nm - 530nm) Standard mode	Active, Gain: 100, Offset: 0
HyD Timegate (499 nm)	Active, Gate Start: 0.3 ns, Gate End: 6 ns
PMT 2 (590nm - 595nm)	Inactive, Gain: 0, Offset: 0
HyD 3 (595nm - 600nm) Standard mode	Inactive, Gain: 100, Offset: 0
HyD Timegate (-1 nm)	Inactive, Gate Start: 0.3 ns, Gate End: 6 ns
PMT 4 (600nm - 605nm)	Inactive, Gain: 0, Offset: 0
PMT Trans	Inactive, Gain: 0, Offset: 0
Laser Line (405 nm)	Shutter: off, Intensity: 0.0000%
Laser Line (499 nm)	Shutter: on, Intensity: 3.0012%
Laser Line (598 nm)	Shutter: on, Intensity: 0.0000%
Laser Line (653 nm)	Shutter: on, Intensity: 0.0000%
Laser Line (471 nm)	Shutter: on, Intensity: 0.0000%

Laser Line (472 nm)	Shutter: on, Intensity: 0.0000%
Laser Line (668 nm)	Shutter: on, Intensity: 0.0000%
Laser Line (667 nm)	Shutter: on, Intensity: 0.0000%
Laser Line (666 nm)	Shutter: on, Intensity: 0.0000%

Sequential Setting Nr.3

Pinhole	56.6 µm
PinholeAiry	999.82 mAU
EmissionWavelength for PinholeAiry Calculation	580 nm
Target Slider	Target Park
X2 Lens Changer	CS2 UV Optics 2
HyD 1 (609nm - 630nm) Standard mode	Active, Gain: 100, Offset: 0
HyD Timegate (598 nm)	Active, Gate Start: 0.3 ns, Gate End: 6 ns
PMT 2 (641nm - 646nm)	Inactive, Gain: 0, Offset: 0
HyD 3 (646nm - 651nm) Standard mode	Inactive, Gain: 100, Offset: 0
HyD Timegate (-1 nm)	Inactive, Gate Start: 0.3 ns, Gate End: 6 ns
PMT 4 (651nm - 656nm)	Inactive, Gain: 0, Offset: 0
PMT Trans	Inactive, Gain: 0, Offset: 0
Laser Line (405 nm)	Shutter: off, Intensity: 0.0000%
Laser Line (499 nm)	Shutter: on, Intensity: 0.0000%
Laser Line (598 nm)	Shutter: on, Intensity: 0.3999%
Laser Line (653 nm)	Shutter: on, Intensity: 0.0000%
Laser Line (472 nm)	Shutter: on, Intensity: 0.0000%
Laser Line (473 nm)	Shutter: on, Intensity: 0.0000%
Laser Line (666 nm)	Shutter: on, Intensity: 0.0000%
Laser Line (665 nm)	Shutter: on, Intensity: 0.0000%
Laser Line (664 nm)	Shutter: on, Intensity: 0.0000%

Sequential Setting Nr.4

Pinhole	56.6 µm
PinholeAiry	999.82 mAU
EmissionWavelength for PinholeAiry Calculation	580 nm
Target Slider	Target Park
X2 Lens Changer	CS2 UV Optics 2
HyD 1 (603nm - 641nm) Standard mode	Inactive, Gain: 100, Offset: 0
HyD Timegate (-1 nm)	Inactive, Gate Start: 0.3 ns, Gate End: 6 ns
PMT 2 (641nm - 646nm)	Inactive, Gain: 0, Offset: 0
HyD 3 (661nm - 679nm) Standard mode	Active, Gain: 100, Offset: 0
HyD Timegate (653 nm)	Active, Gate Start: 0.3 ns, Gate End: 6 ns
PMT 4 (775nm - 780nm)	Inactive, Gain: 0, Offset: 0
PMT Trans	Inactive, Gain: 0, Offset: 0
Laser Line (405 nm)	Shutter: off, Intensity: 0.0000%
Laser Line (499 nm)	Shutter: on, Intensity: 0.0000%

Laser Line (598 nm)	Shutter: on, Intensity: 0.0000%
Laser Line (653 nm)	Shutter: on, Intensity: 1.0005%
Laser Line (473 nm)	Shutter: on, Intensity: 0.0000%
Laser Line (474 nm)	Shutter: on, Intensity: 0.0000%
Laser Line (664 nm)	Shutter: on, Intensity: 0.0000%
Laser Line (663 nm)	Shutter: on, Intensity: 0.0000%
Laser Line (662 nm)	Shutter: on, Intensity: 0.0000%
